# Gross Motor Skills Training Leads to Increased Brain-Derived Neurotrophic Factor Levels in Healthy Older Adults: A Pilot Study

**DOI:** 10.3389/fphys.2019.00410

**Published:** 2019-04-12

**Authors:** Catherine-Alexandra Grégoire, Nicolas Berryman, Florence St-Onge, Thien Tuong Minh Vu, Laurent Bosquet, Nathalie Arbour, Louis Bherer

**Affiliations:** ^1^Montreal Heart Institute, Montreal, QC, Canada; ^2^Centre de Recherche de l’Institut Universitaire de Gériatrie de Montréal, Montreal, QC, Canada; ^3^Département de Médecine, Université de Montréal, Montreal, QC, Canada; ^4^Department of Sports Studies, Bishop’s University, Sherbrooke, QC, Canada; ^5^Department of Neuroscience, Université de Montréal, CRCHUM, Montreal, QC, Canada; ^6^Department of Medicine, Centre Hospitalier de l’Université de Montréal, Service de Gériatrie, Montreal, QC, Canada; ^7^Laboratory MOVE (EA 6314), Faculty of Sport Sciences, Université de Poitiers, Poitiers, France; ^8^Department of Kinesiology, Université de Montréal, Montreal, QC, Canada; ^9^PERFORM Centre, Concordia University, Montreal, QC, Canada

**Keywords:** aging, exercise, fitness, biomarkers, cognition

## Abstract

Exercise is recognized as a promising approach to counteract aging-associated declines in cognitive functions. However, the exact molecular pathways involved remain unclear. Aerobic training interventions and improvements in peak oxygen uptake (VO_2_peak) have been associated with increases in the peripheral concentration of brain-derived neurotrophic factor (BDNF) and better cognitive performances. However, other training interventions such as resistance training and gross motor skills programs were also linked with improvements in cognitive functions. Thus far, few studies have compared different types of physical exercise training protocols and their impact on BDNF concentrations, especially in participants over 60 years old. The main objective of this study was to compare the effects of three exercise protocols on plasma BDNF concentrations at rest in healthy older adults. Thirty-four older adults were randomized into three interventions: (1) lower body strength and aerobic training (LBS-A), (2) upper body strength and aerobic training (UBS-A), or (3) gross motor activities (GMA). All interventions were composed of 3 weekly sessions over a period of 8 weeks. Physical, biochemical, and cognitive assessments were performed pre and post-intervention. All interventions resulted in improved cognitive functions but the GMA intervention induced a larger increase in plasma BDNF concentrations than LBS-A. No correlation was observed between changes in BDNF concentrations and cognitive performances. These findings suggest that a program of GMA could lead to enhancements in plasma BDNF concentrations. Moreover, cognition improvement could occur without concomitant detectable changes in BDNF, which highlights the multifactorial nature of the exercise-cognition relationship in older adults.

## Introduction

Studies support the benefits of physical exercise on cognition in older adults (Kramer and Colcombe, 2018). Mediators of this relationship can include cellular and molecular changes, structural and functional brain adaptations (Stillman et al., 2016). Exercise-mediated augmentation of growth factors, such as the brain-derived neurotrophic factor (BDNF), could play an important role.

Brain-derived neurotrophic factor is a member of the neurotrophin family that acts on neuronal survival, growth and maintenance (Bowling et al., 2016; Begni et al., 2017). While BDNF is found throughout the entire brain, higher concentrations are reported in the hippocampus, cerebral cortex, and cerebellum (Murer et al., 2001; Erickson et al., 2012). Notably, variations of plasma BDNF concentrations in humans paralleled those detected in cerebrospinal fluid (Pillai et al., 2010). Importantly, older adults presenting higher plasma BDNF concentrations perform better on executive functions and verbal fluency tests and show less functional impairments (Vaughan et al., 2014; Navarro-Martinez et al., 2015).

Studies suggest that aerobic physical exercise can boost BDNF concentration (Erickson et al., 2011; Heijnen et al., 2015; Szuhany et al., 2015). Whether specific physical training protocols exhibit greater impact on BDNF production is still undetermined. This question is paramount given that a gross motor skills intervention resulted in similar cognitive enhancements than a combined strength and aerobic training regimen (Berryman et al., 2014). The objective of this pilot study was to investigate the plasma BDNF response to these different training interventions. We hypothesized that interventions with an aerobic component would lead to greater BDNF concentrations than a gross motor skills program and that variations in BDNF concentrations would positively correlate with changes in cognitive performances.

## Materials and Methods

### General Overview

The actual report is a sub-study of a published paper (Berryman et al., 2014). The participants were randomized into three different interventions: (1) high-intensity aerobic and strength training of the lower body (LBS-A), (2) high-intensity aerobic and strength training of the upper body (UBS-A), and (3) gross motor activities (GMA). Tests were conducted before and after an 8-week intervention. Protocol and consent forms were approved by the Research Ethics Board of the Montreal Geriatric Institute.

### Participants

Participants were between 60 and 85 years old. Exclusion criteria included: uptake of medication affecting gait and balance; diagnosis of an orthopedic, neurological, cardiovascular, respiratory problem, somatic or psychiatric disease; general anesthesia within 6 months prior to the beginning of the study; restricted mobility, movement disorders, epilepsy; major visual or hearing impairments; smoking, uncontrolled alcohol or drug abuse; and a score below 24 for the Mini Mental State Examination. A total of 47 healthy participants completed at least 85% of all training sessions. Nine participants declined to provide blood samples. Three participants did not fast and were eliminated from the analyses. One participant was later diagnosed with major depression and was excluded. Total sample was composed of 34 participants, 8 women and 5 men (*n* = 13) in LBS-A, 4 women and 7 men (*n* = 11) in UBS-A and 7 women and 3 men (*n* = 10) in GMA.

### Intervention

Participants were randomly assigned to one of the three intervention groups consisting of thrice-weekly 60-min sessions for 8 weeks. After a warm-up, LBS-A participants began each training session with resistance exercises for the lower body (leg press, leg curl, leg extension, standing plantar flexion, hip extension), while UBS-A participants did resistance exercises focused on the upper body (seated chest press, shoulder frontal and lateral abductions, shoulder external rotations, wrist flexion, horizontal rowing). For both LBS-A and UBS-A participants, this section was followed by an aerobic training on a cycle ergometer. On Mondays and Fridays, LBS-A and UBS-A participants were exposed to high-intensity interval training, including two sets of 4 to 7 min separated by a passive recovery period of 5 min. On Wednesdays, LBS-A and UBS-A participants went through a continuous 20-min aerobic training. For GMA participants, the warm-up was followed by exercises involving static and dynamic stretching, locomotion (walking through obstacles), coordination (juggling and ball-throwing exercises toward a target) and relaxation (slow breathing patterns).

### Tests and Measures

A complete description of tests and measures (i.e., neuropsychological battery, the random number generation (RNG) task, and VO_2_peak) is detailed in Berryman et al. (2014).

#### Laboratory Data

All blood draws were performed following a 12-h fasting period in a resting state in the morning and timing was consistent between pre- and post-testing sessions. Blood samples were collected in EDTA K2-coated tubes and centrifuged to collect plasma. Samples were kept in -80°C freezers for 2–3 years before BDNF analyses. Plasma BDNF concentrations were measured in duplicates, and samples from the same donor (pre-post) were run on the same plate with the Human BDNF enzyme-linked immunosorbent assay (Quantikine ELISA Kit; R&D Systems, Minneapolis, MN, United States) according to the manufacturer’s instructions. These assays have intra-assay coefficients of variation between 3.8 and 6.2%. The technician was blinded to participants’ group assignment when completing ELISA assay.

### Statistical Analysis

Baseline differences between groups in neuropsychological tests were assessed with one-way ANOVAs for all variables showing a normal distribution and homogeneity of the variance, followed by Tukey’s *post hoc* test when the null hypothesis was rejected. Otherwise, between group differences were assessed with the Kruskal-Wallis test. Training-related effects on BDNF and VO_2_peak were tested with an ANCOVA on post-test measures using baseline assessment for each measure as a covariate. *Post hoc* pairwise comparisons were done if the effect of the intervention was significant. As was done in the main study, a two-way ANOVA with repeated measures was used for RNG measures (Berryman et al., 2014). When an interaction was found, relative differences were compared between groups using one-way ANOVAs. Due to the skewed distribution of BDNF concentrations, log10 transformed values were used in the analyses. Effect sizes (Hedges’ *g*) were also calculated when significant differences between groups were observed. The non-parametric Spearman’s rank-order correlation was performed to measure the strength and direction of the association between changes in plasma BDNF concentrations and RNG scores. Statistical analyses were performed using SPSS 24 (Chicago, IL). Results were considered statistically significant when *p* ≤ 0.05.

## Results

### General Characteristics

There was no difference between groups in general characteristics at pre-test (see Table 1). GMA participants’ performance level was lower than both LBS-A and UBS-A groups in some neuropsychological tests, including Digit-span forward and backward, Stroop inhibition and flexibility, but all results remained within healthy thresholds based on their age group normative data (Strauss et al., 2006).

**Table 1:** General characteristics of participants.

Pre-testing general characteristics	LBS-A (*n* = 13)		UBS-A (*n* = 11)		GMA (*n* = 10)	
Age (years)	70.45 (4.44)		70.62 (6.35)		74.59 (5.18)	
Gender	F (8) M (5)		F (4) M (7)		F (7) M (3)	
Education (years)	14.46 (3.64)		14.00 (3.03)		14.90 (2.73)	
MMSE (score/30)	28.85 (1.28)		28.36 (1.21)		29.30 (0.82)	
GDS (score/30)	3.15 (3.26)		3.09 (2.88)		3.50 (3.92)	
**Neuropsychological assessments**
*Verbal reasoning:*						
Similarities (score/32)	23.38 (5.27)		20.73 (6.20)		17.60 (4.84)	
*Short-term memory:*						
Digit-span (forward) total score/16	10.92 (1.98)^b0.014^		9.55 (2.50)		8.50 (1.35)	
*Working memory:*						
Digit-span (backward) total score/14	7.92 (2.25)^a0.042^		6.18 (2.09)		5.70 (1.83)	
*Processing speed:*						
Coding (score/132)	62.54 (13.77)		55.09 (16.14)		58.90 (12.12)	
Stroop color (s)	29.68 (5.28)		33.63 (5.79)		35.13 (8.72)	
Stroop reading (s)	20.81 (3.67)		23.38 (2.73)		22.95 (4.33)	
Trail A (s)	42.69 (10.49)		37.95 (13.67)		45.47 (8.72)	
*Executive functions:*						
Stroop inhibition (s)	57.69 (9.23)^a0.002^		64.13 (11.61)		75.52 (12.41)	
Stroop inhibition/flex (s)	59.35 (8.72)^b0.006^		69.21 (7.92)		78.19 (19.29)	
Trail B (s)	105.13 (40.50)		98.69 (69.08)		107.35 (39.99)	

**Plasma BDNF concentrations and aerobic fitness**

	**LBS-A (*n* = 13)**		**UBS-A (*n* = 11)**		**GMA (*n* = 10)**	
	**Pre**	**Post**	**Pre**	**Post**	**Pre**	**Post**

BDNF (pg/mL)	1108.42	388.13	1220.42	688.27	694.53	1886.38
	(1366.55)	(441.48)	(1708.00)	(641.69)	(864.66)	(1545.97)
Log BDNF ^∗∗^ (pg/mL)	2.56	2.33	2.59	2.62	2.48	3.03
	(0.76)	(0.49)	(0.72)	(0.49)	(0.63)	(0.58)
VO_2_peak ^∗∗^/^∗∗∗^(mL kg^-1^ min^-1^)	26.72	28.84	26.17	29.26	18.86	19.04
	(4.36)	(4.63)	(6.27)	(5.80)	(4.02)	(4.15)


*Values are reported as mean (SD) or number of participants. ^*a*^*p*-value, different from GMA at baseline, Tukey’s *post hoc* test. ^*b*^*p*-value, different from GMA at baseline, Kruskal-Wallis. ANCOVA (baseline values as covariate). ^∗∗^*p* = 0.002 LBS-A vs. GMA (post; BDNF); *p* = 0.007 LBS-A vs. GMA (post; VO_*2*_peak). ^∗∗∗^*p* ≤ 0.001 UBS-A vs. GMA (post; VO_*2*_peak)*.

### Plasma BDNF Concentration

Log10 transformed plasma BDNF concentrations were compared amongst all three groups (Table 1). The ANCOVA showed a significant effect of the 8-week intervention on plasma BDNF concentrations [*F*_(2,31)_ = 5.500, *p* = 0.009]. The following *post hoc* pairwise comparisons further demonstrated that the difference at post-test between the LBS-A group and GMA was significant (*p* = 0.002).

### Aerobic Fitness

VO_2_peak values are presented in Table 1. As expected, an ANCOVA analysis [*F*_(1,32)_ = 7.724, *p* = 0.002] showed that LBS-A and UBS-A interventions led to greater aerobic improvements than the GMA intervention (LBS-A vs. GMA: *p* = 0.007, *g* = -2.21; UBS-A vs. GMA: *p* ≤ 0.001, *g* = -2.01).

### Cognition

Results of the RNG task (Table 2) for the present samples replicated those of the main study (Berryman et al., 2014). In single-task, an effect of time for two indices of inhibition (Turning point index: *F*_(1,31)_ = 5.383, *p* = 0.027; and adjacency index: *F*_(1,31)_ = 6.398, *p* = 0.017) was found. The magnitude of the pre-post difference was considered small for turning point index (*g* = 0.443, 0.227, 0.399 for LBS-A, UBS-A, and GMA, respectively) and for adjacency index (*g* = -0.547, -0.171, -0.334 for LBS-A, UBS-A, and GMA, respectively).

**Table 2:** Random number generation performance.

	LBS-A (*n* = 13)		UBS-A (*n* = 11)		GMA (*n* = 10)	
	Pre	Post	Pre	Post	Pre	Post
**Inhibition at rest (single task)**
TPI^a0.027^	82.12 (13.10)	87.56 (11.40)	71.24 (13.19)	73.82 (9.16)	79.42 (20.27)	85.71 (9.34)
Runs	1.02 (0.43)	1.07 (0.28)	1.51 (0.65)	1.42 (0.57)	1.09 (0.62)	0.88 (0.30)
Adjacency^a0.017^	34.85 (8.32)	30.06 (9.16)	44.77 (10.19)	43.05 (9.94)	37.64 (15.33)	33.50 (8.53)
**Working memory at rest (single task)**
R	1.81 (1.49)	1.43 (0.74)	1.46 (0.75)	1.68 (0.92)	1.46 (0.69)	1.90 (0.83)
Coupon	18.05 (4.31)	17.23 (2.77)	17.49 (2.75)	18.04 (5.15)	17.06 (2.93)	17.47 (3.98)
MRG	7.58 (0.70)	7.73 (0.44)	7.91 (0.30)	7.68 (0.46)	7.65 (0.47)	7.45 (0.28)
**Inhibition while walking at 4 km/h (dual task)**
TPI	83.36 (13.67)	84.77 (15.91)	71.10 (16.83)	64.70 (13.06)	83.57 (18.43)	85.26 (15.17)
Runs	1.13 (0.52)	1.21 (0.49)	1.80 (0.75)	1.62 (0.84)	1.16 (0.88)	0.77 (0.30)
Adjacency^a0.071^	34.85 (12.43)	32.23 (9.95)	47.27 (11.91)	47.55 (11.96)	39.30 (16.47)	32.40 (10.08)
**Working memory while walking at 4 km/h (dual task)**
R^∗^	1.87 (0.98)	1.22 (0.56)	1.69 (1.15)	1.82 (1.31)	1.65 (0.62)	2.37 (1.48)
Coupon	17.63 (4.66)	15.81 (1.96)	20.45 (8.36)	18.81 (8.42)	17.80 (3.62)	20.87 (5.02)
MRG	7.54 (0.78)	7.77 (0.60)	7.55 (0.52)	7.64 (0.67)	7.60 (0.84)	7.30 (0.67)


*Values are reported as mean (SD). TPI: turning point index; R: redundancy index; MRG: mean repetition gap. For runs, adjacency, R, and coupon scores, lower scores represent better performances. ^*a*^*p*-value, time effect. Interaction: ^∗^*p* = 0.019*.

In dual-task, the RNG task was performed while walking at 4 km h^-1^. A trend for an effect of time was found (*p* = 0.071) for the adjacency index. In addition, a time by group interaction for the working memory redundancy index [*F*_(2,31)_ = 4.493, *p* = 0.019] was observed, but no significant difference in the pre-post relative changes on the redundancy index was detected. The magnitude of the pre-post difference was considered trivial to moderate for the adjacency index (*g* = -0.233, 0.023, -0.505 for LBS-A, UBS-A, and GMA, respectively) and trivial for redundancy in the UBS-A group (*g* = 0.105). However, the magnitude was considered moderate to large for redundancy in both the LBS-A (*g* = -0.814) and GMA (*g* = 0.635) groups.

### Changes in Cognitive Performances and BDNF Concentrations

There was no significant correlation between changes in BDNF concentrations and performance changes in the single RNG task (Turning point, LBS-A: *r* = 0.225, *p* = 0.459; UBS-A: *r* = 0.127, *p* = 0.709; GMA: *r* = 0.018, *p* = 0.960 and Adjacency, LBS-A: *r* = 0.418, *p* = 0.156; UBS-A: *r* = 0.009, *p* = 0.979; GMA: *r* = -0.261, *p* = 0.467), and in the dual RNG task (Redundancy index, LBS-A: *r* = -0.060, *p* = 0.845; UBS-A: *r* = 0.391, *p* = 0.235; GMA: *r* = 0.285, *p* = 0.425).

## Discussion

Contrary to our main hypothesis, GMA induced a bigger increase in plasma BDNF concentrations than interventions with an aerobic component. In addition, no correlation was observed between changes in BDNF concentrations and changes in RNG indices.

Despite a limited amount of studies available on gross motor skills training, our data are consistent with a report demonstrating that BDNF levels can be improved after a variety of training protocols. Indeed, Pal et al. (2014) detected a significant increase in plasma BDNF levels in 20–50 years old participants following yoga sessions, consisting of complex stretching and breathing exercises, included in our GMA routine.

Results reported here do not show an association between BDNF concentrations and cognition as assessed with the RNG task. This supports the notion that multiple pathways could explain improvements in cognition observed after an exercise protocol (Stillman et al., 2016) and that BDNF changes might only be one factor among others potentially supporting cognitive adaptations following an exercise program.

Nevertheless, the absence of a significant increase in BDNF following an aerobic training program in the present study is equivocal (Cassilhas et al., 2012). A study reported that relative changes in BDNF, VO_2_peak and memory performance were associated with changes in hippocampus volume after a 12-month brisk-walking intervention with older adults (Erickson et al., 2011). Therefore, since our intervention led to similar changes in VO_2_peak (+7.8% vs. +7.9% for LBS-A and +11.8% for UBS-A), it appears that training load parameters (type of exercise, protocol duration and weekly volume) might be more important than changes in VO_2_peak, at least with regard to the BDNF molecular pathway and its relationship with cognitive improvements in older adults. Our cycling program combined with resistance training had a lower training volume (8 vs. 52 weeks; 36–48 vs. 120 min weekly). Future studies should measure BDNF levels both acutely post-training and at rest to better capture the impact of training.

This pilot study has limitations. First, we acknowledge the absence of a control group, and we cannot rule out a potential effect of regression to the mean. Second, small sample size, lack of gender comparisons and absence of genotyping can influence BDNF (Barha et al., 2017). Lower plasma BDNF concentrations were reported in women than in men (Lommatzsch et al., 2005). The single nucleotide polymorphism (SNP) in BDNF sequence leading to a methionine (Met) instead of valine (Val) residue (Val66Met) is associated with diminished BDNF secretion levels (Egan et al., 2003; Begni et al., 2017). Notably, a treadmill exercise program led to increased BDNF concentrations in most individuals but not in Val66Met young male carriers, suggesting that the polymorphism negatively alters peripheral BDNF concentration (Lemos et al., 2016). In contrast, another group reported that in adults above 50 years old, although Val66Met carrier men had lower BDNF concentrations than those not carrying this SNP, women had similar levels regardless of the SNP (Bus et al., 2012). Therefore, future studies should include large cohorts of participants to address such impacts on elderly adults enrolled in different exercise programs.

## Conclusion

This study supports the notion that exercise programs with a focus on gross motor skills development could result in elevated BDNF concentrations in healthy older adults. Furthermore, improvements in cognitive performances could be observed without significant concomitant increases in BDNF levels.

## Ethics Statement

This study was approved and carried out in accordance with the recommendations of the ethics guidelines of the Comité d’éthique de la recherche vieillissement-neuroimagerie du CIUSSS du Centre-Sud-de-l’île-de-Montréal with written informed consent from all subjects. All subjects gave written informed consent in accordance with the Declaration of Helsinki.

## Author Contributions

NB, LBo, and LBh conceived and designed the experiments. NB, FSt-O, and TV performed the tests. C-AG, NB, FSt-O, and NA analyzed the data. C-AG, NB, and LBh wrote the manuscript. All authors revised and contributed to the manuscript for important intellectual content.

## Conflict of Interest Statement

The authors declare that the research was conducted in the absence of any commercial or financial relationships that could be construed as a potential conflict of interest.
